# Supplementary material for: Antibody Levels at 3-Years Follow-Up of a Third Dose of Measles-Mumps-Rubella Vaccine in Young Adults
Source: Vaccines (Basel). 2022 Jan 17;10(1):132. doi: 10.3390/vaccines10010132 (PMC8781586; doi:10.3390/vaccines10010132)
Supplement: Supplementary file 1 [file vaccines-10-00132-s001.zip › vaccines-1531568-supplementary.pdf]

Supplementary Materials

Belonging to original Research Manuscript:

Antibody levels at 3-years follow-up of a third dose of measles-mumps-rubella vaccine in young adults

Patricia Kaaijk, Alienke J. Wijmenga-Monsuur, Hinke I. ten Hulscher, Jeroen Kerkhof, Gaby Smits, M. Alina Nicolaie, Marianne A. van Houten, Rob S. van Binnendijk

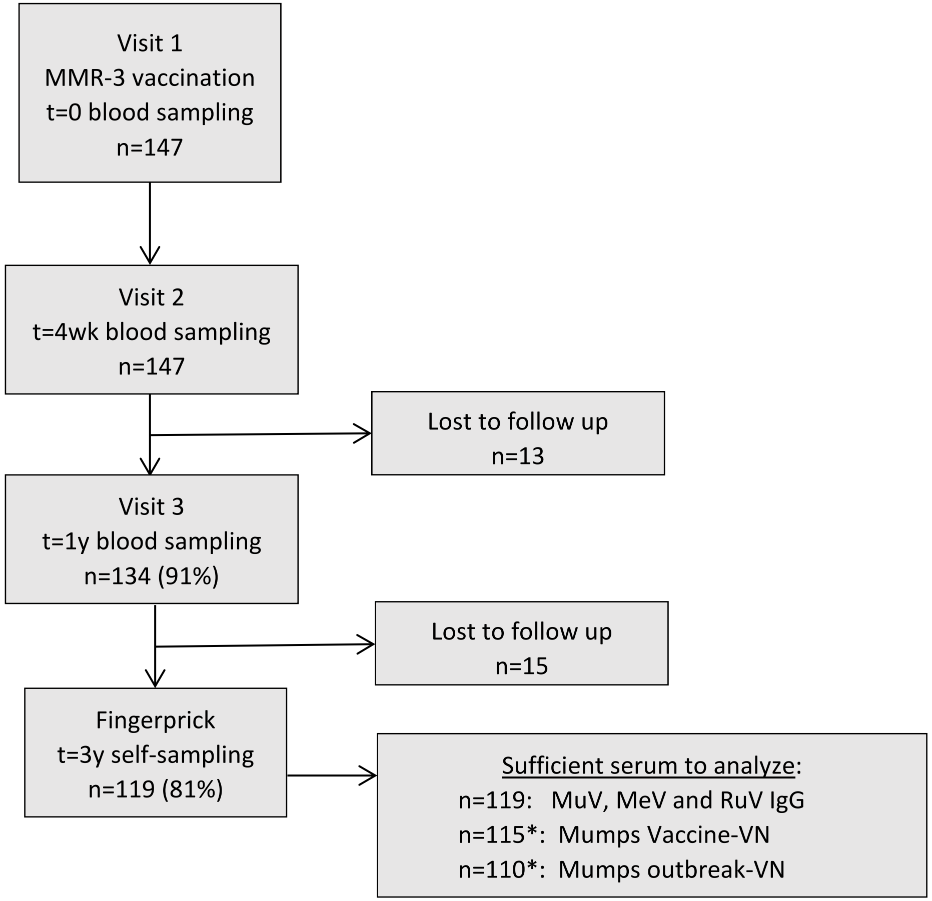

Supplementary Figure S1. Flow diagram for blood sampling of study participants.

Number of participants with blood samples collected during visit 1 (pre-vaccination, t=0), visit 2 (4 weeks post-vaccination) and visit 3 (1 year post-vaccination). Collected blood sample volume was sufficient to perform all antibody analyses for all vaccine components. Fingerprick sample volumes at 3 years post-vaccination (n=119) was sufficient to perform all IgG analyses for measles virus (MeV), mumps virus (MuV), and rubella virus (RuV), but there was not sufficient volume of serum left for all mumpsvirus neutralization (VN) assays, as indicated in flow diagram*.

Supplementary Table S1. Geomean IgG concentrations, and ND50 values of antibodies against mumps virus at various time points from an MMR3 dose.

	**Mumps IgG**				**Mumps Vaccine-VN**				**Mumps outbreak-VN**			
**MMR3**	**Pre-MMR3 n=147**	**4wk n=147**	**1y n=134**	**3y n=119**	**Pre- MMR3 n=147**	**4wk n=147**	**1y n=134**	**3y n=115***	**Pre- MMR3 n=147**	**4wk n=147**	**1y n=134**	**3y n=110***
GMC IgG (RU/mL) or ND_{50} [95% CI]	185 [163-211]	306 [273-343]	255 [224-290]	228 [200-259]	84.3 [68.3-104]	115 [95.8-139]	102 [84.6-123]	93.8 [76.7-115]	63.8 [52.7-77.2]	86.6 [73.2-103]	82.8 [70.4-97.5]	82.8 [67.9-101]
Seroprotection	81%	94%	90%	87%	78%	86%	86%	82%	78%	88%	89%	85%

Data are presented as geometric mean (GMC) of IgG concentrations in RU/mL and virus neutralizing antibodies (VN) in 50% neutralization dose (ND_{50}) [95% confidence interval].
Seroprotection is defined as proportion of participants with antibody concentrations above specified cutoff levels for protection against mumps, with a cutoff level of 102 RU/ml, ND_{50} of 34 and ND_{50} of 26 for respectively mumps IgG concentration, virus neutralizing antibodies against mumps virus vaccine strain and virus neutralizing antibodies against mumps virus outbreak strain
Abbreviations: Pre-MMR3, prior to a third dose of measles-mumps-rubella vaccine dose (MMR3); 4wk, 1y and 3 y, respectively 4 weeks, 1 year and 3 years after an MMR3; MuV, mumps virus; RU/mL, RIVM units per milliliter; vaccine-VN, virus neutralizing antibodies against mumps virus vaccine strain; outbreak-VN, virus neutralizing antibodies against mumps virus outbreak strain; ND_{50} values, virus-neutralizing antibody titer which resulted in 50% plaque reduction

* Dependent on the available amount of serum from finger prick, not all VN data could be determined for sera of all participants at 3 years

Supplementary Table S2. Geomean concentrations of IgG antibodies against measles, mumps, and rubella viruses at various time points from an MMR3 dose.

	**Measles**				**Mumps**				**Rubella**			
**MMR3**	**Pre-MMR3 n=147**	**4wk n=147**	**1y n=134**	**3y n=119**	**Pre- MMR3 n=147**	**4wk n=147**	**1y n=134**	**3y n=119**	**Pre- MMR3 n=147**	**4wk n=147**	**1y n=134**	**3y n=119**
IgG (IU or RU/mL) [95% CI]	0.69 [0.59-0.80]	1.23 [1.10-1.38]	1.04 [0.92-1.17]	0.87 [0.76-1.00]	185 [163-211]	306 [273-343]	255 [224-290]	228 [200-259]	36.7 [32.4-41.6]	111 [100-122]	64.8 [58.1-72.5]	49.0 [43.6-55.2]
Seroprotection	97%	100%	100%	100%	81%	94%	90%	87%	95%	100%	100%	100%

Data are presented as geometric mean IgG concentrations (GMC) [95% confidence interval].
Seroprotection is defined as proportion of participants with antibody concentrations above specified cutoff IgG antibody levels for protection against mumps (102 RU/ml), measles (0.12 IU/ml) or rubella (10 IU/ml) virus
Abbreviations: Pre-MMR3, prior to a third dose of measles-mumps-rubella vaccine dose (MMR3); 4wk, 1y and 3 y, respectively 4 weeks, 1 year and 3 years after an MMR3; MuV, mumps virus; RU/mL, RIVM units per milliliter (used for anti-mumps IgG concentrations); IU/mL, international units per milliliter; IU/mL, international units per milliliter (used for anti-measles and anti-rubella IgG concentrations)
